# NURSING STUDENTS’ RESPONSE TO THE AGE-FRIENDLY HEALTH SYSTEMS 4MS FRAMEWORK CURRICULUM

**DOI:** 10.1093/geroni/igad104.2120

**Published:** 2023-12-21

**Authors:** Timothy O’Connor, Naushira Pandya, Sweta Tewary, Oksana Shnayder

**Affiliations:** Nova Southeastern University, Davie, Florida, United States; Nova Southeastern University, Kiran Patel College of Osteopathic Medicine, Davie, Florida, United States; Nova Southeastern University, Davie, Florida, United States; Nova Southeastern University, Davie, Florida, United States

## Abstract

**Problem:**

There is a shortage of registered nurses, especially those choosing to work in long-term care. Providing interdisciplinary education on age-friendly health care to nursing students offers long-term care as a career option post-graduation. To evaluate the effectiveness of teaching nursing students about age-friendly health care, the students must provide adequate feedback on the educational content they receive. Purpose: This project is part of the Age-Friendly Health Systems 4Ms project and identified why nursing students did not complete a comprehensive survey after receiving an interdisciplinary educational session based on the Age-Friendly Health Systems 4Ms Framework. Research design: A focus group design was used, and subjects were recruited from nursing students who received the educational session but did not complete the comprehensive survey. Questions for the focus group discussion were derived from the content received during the academic session and from the comprehensive survey. The focus group interview was audio-recorded, and the recordings were transcribed verbatim. Data analysis: Focus group transcripts were analyzed using descriptive content analysis to determine themes.

**Results:**

The outcome of this study provided researchers with insight into why nursing students did not complete the comprehensive survey. The results of this study will help improve future comprehensive survey response rates for the remainder of the Age-Friendly Health Systems 4Ms project. Significance: The findings of this study enhances the validity and reliability of the comprehensive survey results and informs researchers across disciplines on how to avoid low survey response rates.

